# *Helicobacter pylori*: Infection and New Perspective for the Treatment

**DOI:** 10.1155/2019/9431369

**Published:** 2019-07-29

**Authors:** Teresa Fasciana, Paola Di Carlo, Ahlem Jouini, Mara Di Giulio

**Affiliations:** ^1^Department of Health Promotion, Mother and Child Care, Internal Medicine and Medical Specialties, University of Palermo, Palermo, Italy; ^2^Laboratory of Epidemiology and Veterinary Microbiology, Group of Bacteriology and Biotechnology Development, Pasteur Institute of Tunis, Université de Tunis El Manar, Tunis, Tunisia; ^3^Department of Pharmacy, “G. d'Annunzio” University Chieti-Pescara, Chieti, Italy

The alarming phenomenon of antibiotic resistance in *Helicobacter pylori* suggests to pay close attention in the treatment. In particular, the clarithromycin resistance in *H. pylori* was designed by the World Health Organization (WHO) as high priority for antibiotic research in 2017 [1]. In Italy, the overall primary resistance to clarithromycin can be detected in 35.2% of cases; in France, it can be detected in 26% of cases, while in Spain, clarithromycin resistance is present in 27.2% of strains [2–4].

Natural/innovative strategies, as well as novel potentiators to restore the antibiotic susceptibility in resistant strains, could be used to improve the efficacy of *H. pylori* eradication, and they could be worthy to change attitude of medicine in dealing with the challenge known as *H. pylori* [5, 6].

Obviously, these therapeutic strategies should be used in patients infected by resistant *H. pylori* and in presence of coinfection with other pathogens responsible to develop severe gastric diseases [7].

On the basis of this evidence, the aim of this special issue was to collect research manuscripts and review manuscripts, case reports, and literature reviews with the objective to expand our knowledge in this innovative field.

In this special issue, a total of seven manuscripts were received, and five of these were accepted.

This issue confirmed that the prevalence of *H. pylori* in developing countries is high because the population lives in households with low socioeconomic status and hygiene. Therefore, in order to improve the diagnostic accuracy, should be recommended the combination of microscopy and PCR assay for effective monitoring of *H. pylori* infection in these endemic areas. PCR is a more sensitive assay to detect *H. pylori* infection than microscopy, and it is not yet considered as the gold standard assay.

Using a mathematical model and the force of infection, it is possible achieved to translate the decreasing pattern into the time-dependent decline in the hazard rate of infection and also permitted the future prediction of seroprevalence in areas with high prevalence of infection. Moreover, the model could be used to predict the future size of gastric cancer.

In experimental studies, it has been observed that the use of 52 kDa *H. pylori* membrane peptide as a vaccine has been effective to immunize against the development of gastric ulcer when used in murine models. However, the isolation and purification of such protein presents important challenges; therefore, the use of synthetic peptides designed from immunogenic proteins has become an alternative for diagnosis and prophylaxis. Since *H. pylori* causes superficial infection of the gastric tissue, the main immunity mediators are secretory IgA antibodies, which are the objective of active oral vaccination. Immunized animals produce specific serum IgG and IgA and intestinal and salivary IgAs, and after challenge, a gastric cellular and antibody response can be observed. One immunogen-derived peptide antigen of 50–52 kDa with the amino-terminal end sequence Met-Val-Thr-Leu-Ile-Asn-Asn-Glu (MVTLINNE) produced by *H. pylori* could be used to the prophylaxis of its infection. The results showed that immunization with the MVTLINNE peptide stimulated the cellular immune response and increased proliferative response of thymus lymphocytes. In addition, MVTLINNE peptide vaccination-mediated IgA production correlated with no alterations in the gastric mucosa and scarce presence of bacilli after *H. pylori* infection. In conclusion, these results indicated that prophylactic immunization significantly reduced the number of colonizing bacteria, which was associated with healthy gastric tissue.

In the era with high percentage of resistance to clarithromycin, rifabutin, furazolidone, and tetracycline, alone or in combination, are promising candidates for rescue therapy of antibiotic-resistant *H. pylori* strains, as no definitive rescue therapy for *H. pylori* eradication is available.

In our opinion, this first year of this special issue attracted interest in a field that is growing development, and we hope that the information contained in this special issue will help to develop new strategies to prevent/treat the *H. pylori* infection.
